# Edges of Layered FePSe_3_ Exhibit Increased Electrochemical and Electrocatalytic Activity Compared to Basal Planes

**DOI:** 10.1021/acsaelm.2c01493

**Published:** 2023-02-13

**Authors:** Stefan Wert, Christian Iffelsberger, Akshay Kumar K. Padinjareveetil, Martin Pumera

**Affiliations:** † Future Energy and Innovation Laboratory, Central European Institute of Technology, Brno University of Technology, Purkyňova 123, Brno 61200, Czech Republic; ‡ Energy Research Institute@NTU (ERI@N), Research Techno Plaza, X-Frontier Block, Level 5, 50 Nanyang Drive, Singapore 637553, Singapore; § New Technologies—Research Centre, University of West Bohemia, Univerzitní 8, Plzeň 30100, Czech Republic; ∥ Department of Medical Research, China Medical University Hospital, China Medical University, No. 91 Hsueh-Shih Road, Taichung 40402, Taiwan

**Keywords:** scanning electrochemical microscopy, 2D materials, transition metal trichalcogenphosphites, iron phosphotriselenide, hydrogen evolution reaction, electrocatalysis, electrochemistry

## Abstract

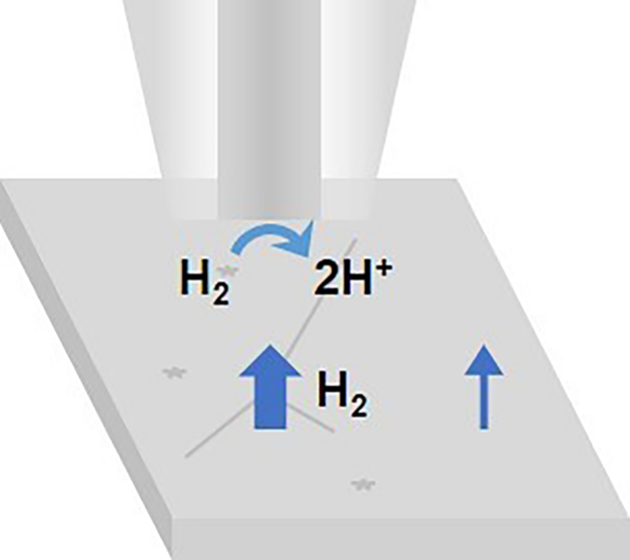

Transition metal trichalcogenphosphites (MPX_3_), belonging to the class of 2D materials, are potentially viable electrocatalysts for the hydrogen evolution reaction (HER). Many 2D and layered materials exhibit different magnitudes of electrochemical and electrocatalytic activity at their edge and basal sites. To find out whether edges or basal planes are the primary sites for catalytic processes at these compounds, we studied the local electrochemical and electrocatalytic activity of FePSe_3_, an MPX_3_ representative that was previously found to be catalytically active. Using scanning electrochemical microscopy, we discovered that electrochemical processes and the HER are occurring at an increased rate at edge-like defects of FePSe_3_ crystals. We correlate our observations using optical microscopy, confocal laser scanning microscopy, scanning electron microscopy, and electron-dispersive X-ray spectroscopy. These findings have profound implications for the application of these materials for electrochemistry as well as for understanding general rules governing the electrochemical performance of layered compounds.

## Introduction

Hydrogen has been identified as a promising medium to store energy and as a green fuel for the transport sector. However, obtaining green hydrogen in an economically feasible manner remains a big challenge. The main green route for producing hydrogen is electrochemical water splitting.^1^ The energy consumption of this process can be decreased using suitable electrocatalysts such as platinum, but its rarity and high cost are the main reasons why electrochemical water splitting cannot be performed on an industrial scale yet.^2^

Consequently, research for alternative catalyst materials has sparked in recent years.^2^ A large group of promising candidates can be found among the so-called 2D materials, which consist of layers of atomic thickness held together by van der Waals forces.^3^ Starting from graphene,^4^ multiple classes of 2D materials have been identified, including, but not limited to, layered pnictogens,^5−8^ transition metal dichalcogenides,^9−13^ transition metal oxides,^14^ metal–organic frameworks,^15,16^ and MXenes.^17−19^ They cover a wide array of remarkable properties such as high electrical conductivity, charge capacity,^20,21^ or electrocatalytic activity^10−13,19,22,23^ depending on the individual material. Another group of 2D materials that has emerged recently is the group of transition metal trichalcogenphosphites (MPX_3_; M: transition metal, P: phosphor, and X: chalcogenide).^24^ They were initially studied mainly for their magnetic properties.^25^ Recent studies however have shown their remarkable electrocatalytic and photocatalytic properties.^26−30^

For the optimization of catalyst materials, it is important to know where the catalytic hotspots are located. It was found that graphite,^31^ graphene,^32−34^ transition metal dichalcogenides,^35−40^ and layered pnictogens^41,42^ show increased electrochemical and electrocatalytic activity at the edges of individual layers compared to their basal planes. In the case of MXenes, the opposite was observed in a study by Djire et al.,^43^ suggesting that the basal planes are more electrocatalytically active. For MPX_3_ compounds, a theoretical study of catalytic hotspots for the cases of FePSe_3_ and MnPSe_3_ was performed.^44^ Based on density functional theory, the authors conclude that edges are more active for the hydrogen evolution reaction (HER) than basal planes. However, these results need to be confirmed by electrochemical experiments. A useful technique to investigate the local electrochemistry of catalysts is scanning electrochemical microscopy (SECM).^45^ It was recently applied for the investigation of the local electrochemical^40^ and electrocatalytic^37,39^ activity of transition metal dichalcogenides. Consequently, the aim of our work was to localize the electrochemical and electrocatalytic hotspots of FePSe_3_ using SECM. We correlated our observations using scanning electron microscopy (SEM) combined with electron-dispersive X-ray spectroscopy (EDS). In addition, X-ray diffraction (XRD) was employed to confirm the composition of the FePSe_3_ sample. For correlating SECM measurements to the sample topography, optical microscopy (OM) and confocal laser scanning microscopy (CLSM) were employed.

## Experimental Section

### Materials and Chemicals

**Figure 1:**
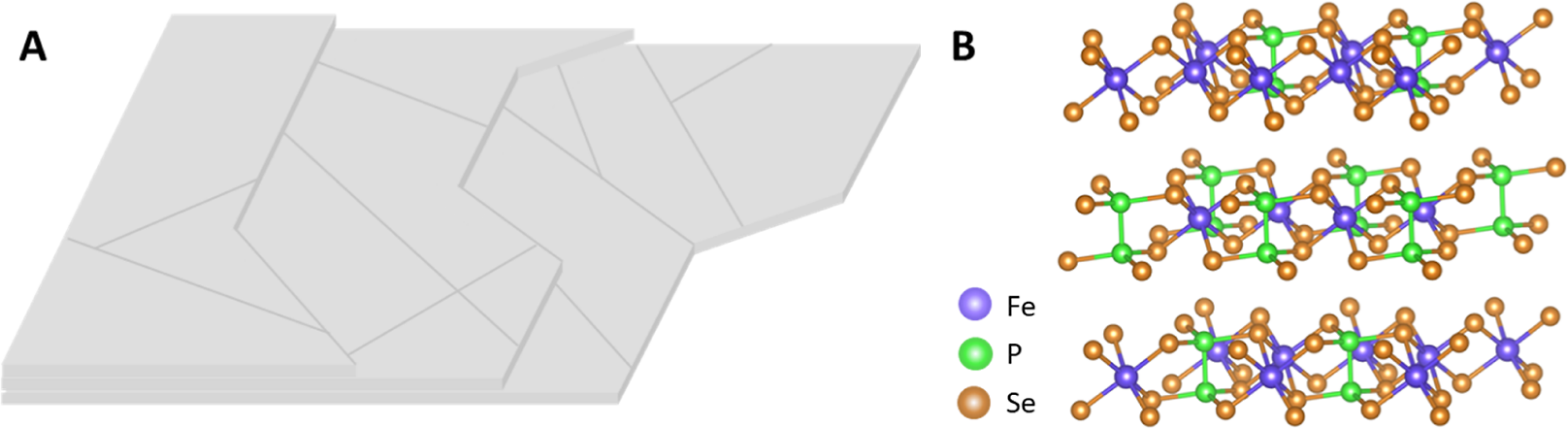
(A) Schematic representation of the surface morphology of a FePSe_3_ crystal. (B) Atomic structure of the FePSe_3_ crystal layers.

Crystalline FePSe_3_ was bought from XFNANO, China. A scheme of the surface morphology of the bulk crystal in given in Figure 1A, with the atomic structure given in Figure 1B. The crystal was prepared according to a previous study^40^ to obtain a flat FePSe_3_ electrode using the following materials. The crystal was embedded in a matrix consisting of a 1:1 (m/m) mixture of two-component epoxy resin (Struers Aps, Denmark) and graphite powder (<20 μm, synthetic, Sigma-Aldrich). Carbon SEM stubs obtained from Micro to Nano, Netherlands, were used as conductive support for the sample. Polydimethylsiloxane (SYLGARD 184) used during the electrode fabrication process was bought from Dow Inc., Michigan, USA. Conductive copper tape was used to establish electrical contact with the sample. All measurements in this work were executed with this sample. Feedback mode and substrate generation/tip collection (SG/TC) mode SECM images were recorded in a solution containing 1.5 mM ferrocene methanol (FcMeOH, 99%, ABCR GmbH, Germany) and 0.2 M potassium nitrate (KNO_3_, analytical grade, Merck KGaA, Germany). For studying the local differences in HER, 0.5 M sulfuric acid (H_2_SO_4_, 96%, analytical grade, Penta, Czech Republic) was used. Deionized water with a resistivity >18.2 MΩ cm (Milli-Q Advantage A10 system, Merck Millipore, Germany) was used to prepare solutions.

### Instrumentation

Localized electrochemical and electrocatalytic activity studies were carried out using a commercially available scanning electrochemical microscope (SECM, Sensolytics, Germany) with a bipotentiostat (PGSTAT302N, Autolab, Netherlands). A 25 μm diameter Pt disk ultramicroelectrode (UME, RG = 11, Sensolytics, Germany) was used for SECM experiments. As counter and reference electrodes for electrochemical measurements, a graphite rod and a Ag/AgCl (3 M KCl) reference electrode were employed. The potentials stated herein refer to this reference system.

Scanning electron micrographs were recorded using a MIRA 3 SEM (Tescan, Czech Republic). For EDS maps, this setup was expanded with a Bruker XFlash 5010 EDS. Accelerating voltages of 5 or 20 kV for SEM and EDS, respectively. Gwyddion 2.55 and Origin 2020 software were used for analyzing and visualizing SECM experiments.

Before recording SECM images, the SECM probe was positioned close to the sample by performing a probe approach curve (PAC) toward the sample surface. The PAC was executed with a probe velocity of 1 μm s^–1^ and a probe potential of *E*_probe_ = 0.5 V. The PAC was stopped when the probe current reached 50% of the current measured in bulk solution. At that distance, SECM images were recorded with a pixel size of 10 μm, a scan rate of 100 μm s^–1^, and a waiting time of 10 ms. Feedback and SG/TC mode images were recorded in a solution of 1.5 mM FcMeOH and 0.2 M KNO_3_. Feedback mode images were obtained with *E*_probe_ = 0.5 V and the substrate at open circuit potential. The SG/TC mode images in FcMeOH were recorded with *E*_probe_ = −0.2 and *E*_substrate_ = 0.5 V, respectively. HER experiments were performed in a solution of 0.5 M H_2_SO_4_. Among these experiments, a linear scan voltammogram was recorded at the FePSe_3_ sample, decreasing the potential from 0.0 to −0.8 V at a scan rate of 5 mV s^–1^. To obtain images of the local catalytic activity of the sample toward the HER, SECM images of the local hydrogen evolution were obtained using potentials of *E*_probe_ = 0.2 V and *E*_substrate_ = −0.5 V. XRD using a diffractometer (SmartLab 3 kW, Rigaku) with a Bragg–Brentano geometry was employed for the structural analysis of the sample. The optical images and height profile measurements of the sample surface were carried out using CLSM (Olympus LEXT OLS4100) with a laser light source (λ = 405 nm).

## Results and Discussion

**Figure 2:**
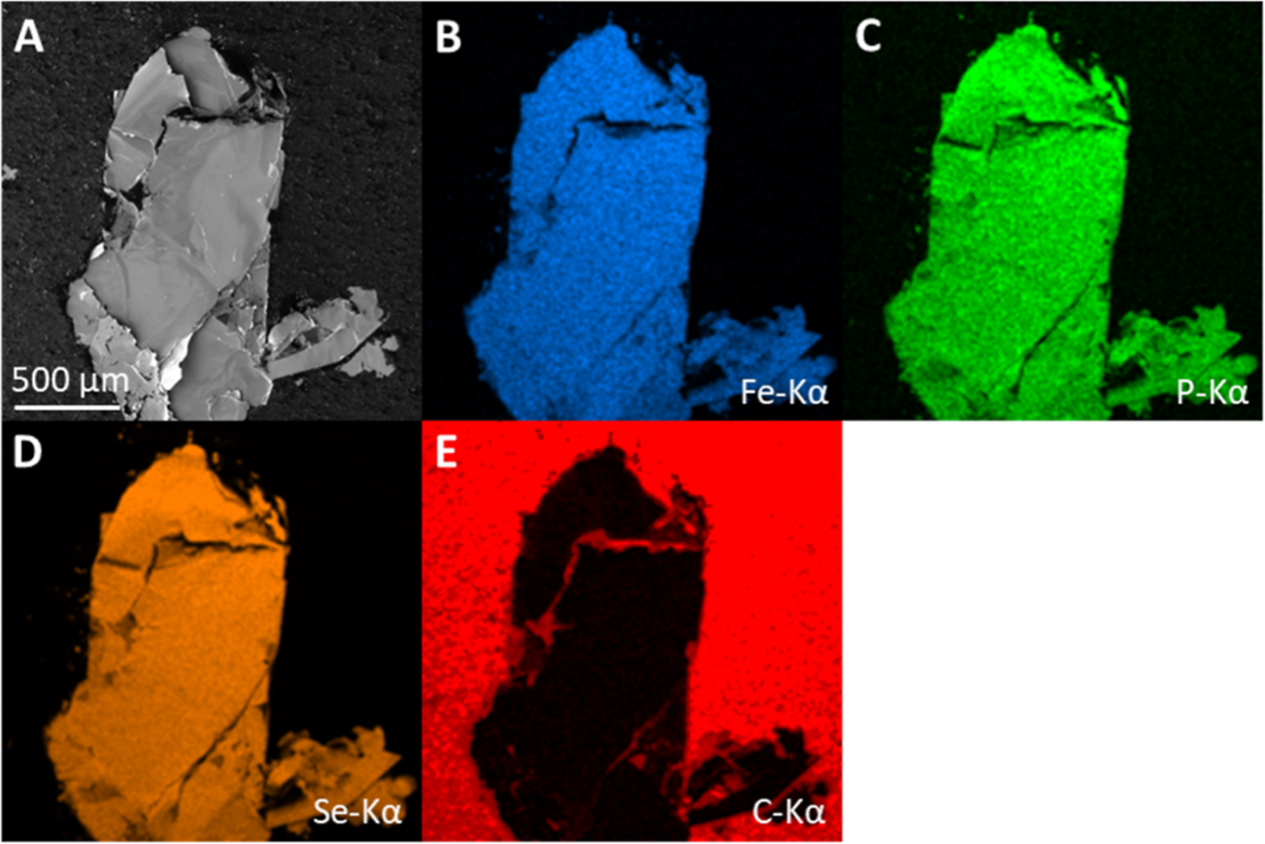
SEM image of the FePSe_3_ sample (A) with EDS elemental maps of Fe (B), P (C), Se (D), and C (E).

**Figure 3:**
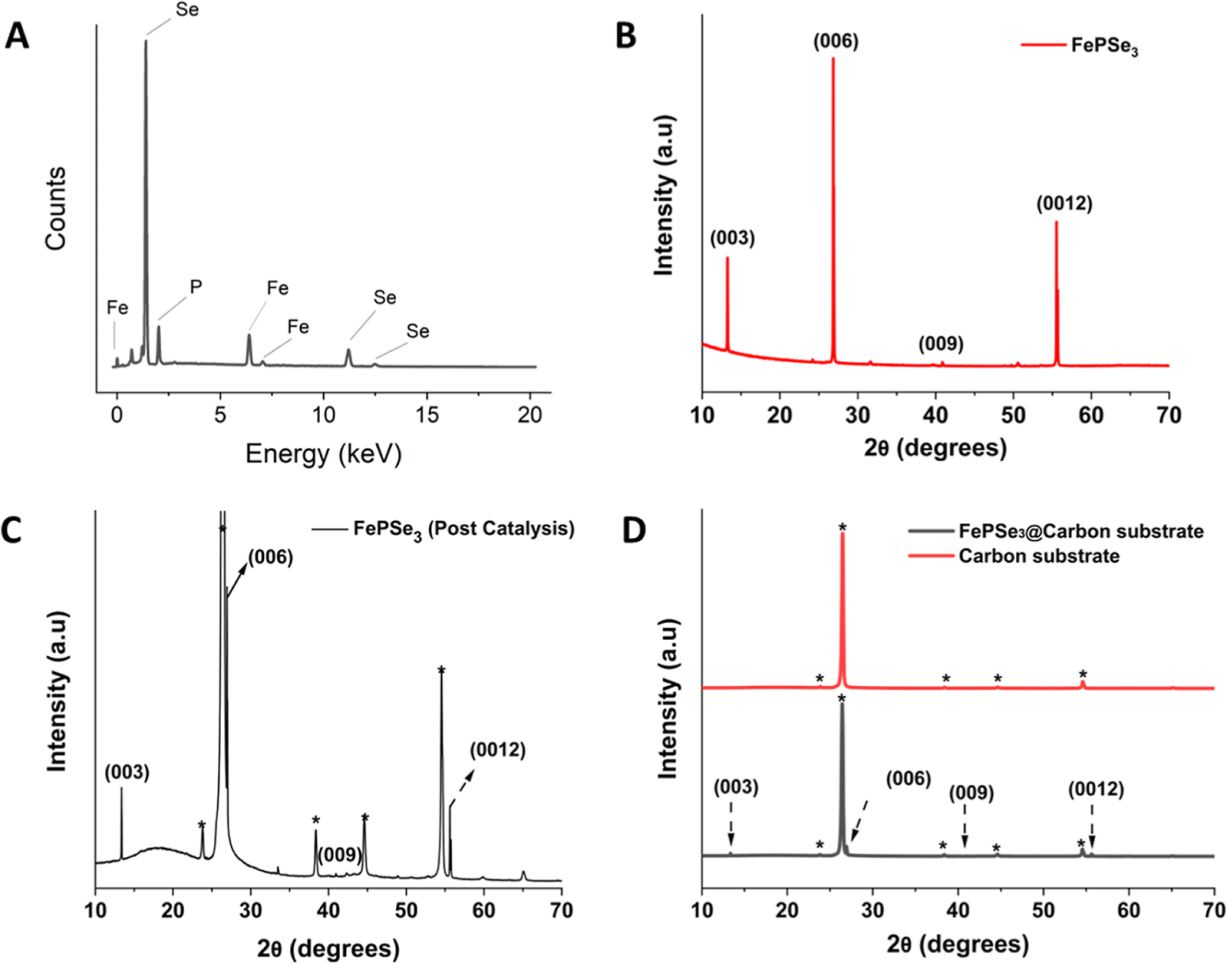
EDS (A) and XRD spectra of FePSe_3_. (B) XRD of the FePSe_3_ crystal prior to sample preparation and HER. (C) XRD spectrum of the crystal embedded in the carbon matrix after HER. (D): Top: XRD of the conductive carbon matrix the FePSe_3_ crystal was embedded into. Bottom: FePSe_3_ sample after HER, embedded in the carbon matrix (same spectrum as in C).

Before electrochemical investigation, an SEM image and EDS elemental maps of the sample were recorded to evaluate the purity and location of the crystal (Figure 2). The SEM image in Figure 2A shows a black area corresponding to the carbon/epoxy matrix surrounding the crystal, which is visible as a gray structure. The EDS elemental maps of Fe, P, and Se (Figure 2B–D, respectively) show a mostly homogeneous distribution within the crystal, with lower amounts detected at large cracks in the sample. Furthermore, local carbon impurities are visible in the crystal (Figure 2E). Nevertheless, most of the crystal surface appears clean. From the EDS spectrum in Figure 3A, atomic percentages for Fe, P, and Se of 21.0%, 19.7%, and 59.3% were derived, indicating high purity of the crystal. The purity of the sample was confirmed by XRD (Figure 3B). The sample gave characteristic crystalline peaks at their respective 2θ value. The values were found to be similar to the ones in the spectrum provided by the crystal manufacturer.^46^ The application of hydrogen evolution to the sample resulted in changes in the relative peak intensity in the FePSe_3_ sample. Figure 3C shows the XRD spectrum of the FePSe_3_ sample after HER. Because the crystal was embedded into a conductive carbon epoxy matrix, the peaks stemming from this matrix are visible as well. They are marked by a *-symbol. XRD spectra of the carbon epoxy matrix and the crystal embedded in said matrix after HER application are given in Figure 3D. Comparing Figure 3B,C, it can be seen that all XRD peaks of the pristine FePSe_3_ crystal are present post-HER as well, and no additional peaks aside the matrix peaks appeared. Thus, it can be assumed that the crystal edges did not undergo reorganization during the HER.

**Figure 4:**
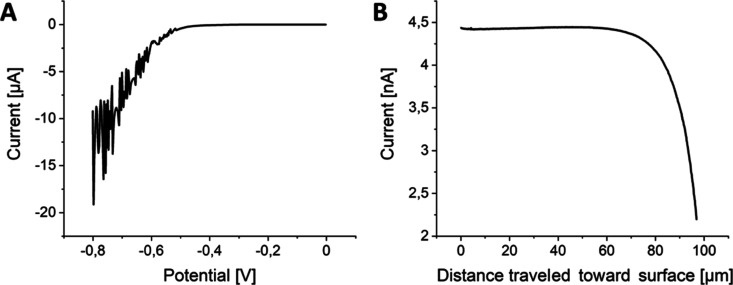
(A) Linear scan voltammogram recorded with the FePSe_3_ sample in 0.5 M H_2_SO_4_ at a scan rate of 5 mV s^–1^. (B) PAC toward the graphite/epoxy region of the FePSe_3_ crystal sample. Parameters: max speed: 1.0 μm s^–1^, step width: 1.0 μm, *E*_probe_ = 0.5 V.

Prior to spatially resolved electrochemical analysis of the FePSe_3_ crystal, an LSV in 0.5 M H_2_SO_4_ was recorded (Figure 4A). Upon scanning toward more negative potentials, the measured current starts decreasing at a potential of −0.5 V due to the beginning of the HER. Decreasing the potential further led to a lower and noisier cathodic current resulting from H_2_ bubble formation at the sample surface. Because a continuous and steady hydrogen evolution is required for SECM imaging, a potential of −0.5 V was applied to the crystal sample during the SECM experiments for imaging the HER.

Before SECM imaging, the probe was brought close to the sample by performing a PAC toward the carbon matrix around the crystal (Figure 4B). As the probe approached the surface, the measured current started to decrease significantly after the probe traveled 60 μm. The approach was stopped when the current decreased by 50% with respect to the initially measured value.

**Figure 5:**
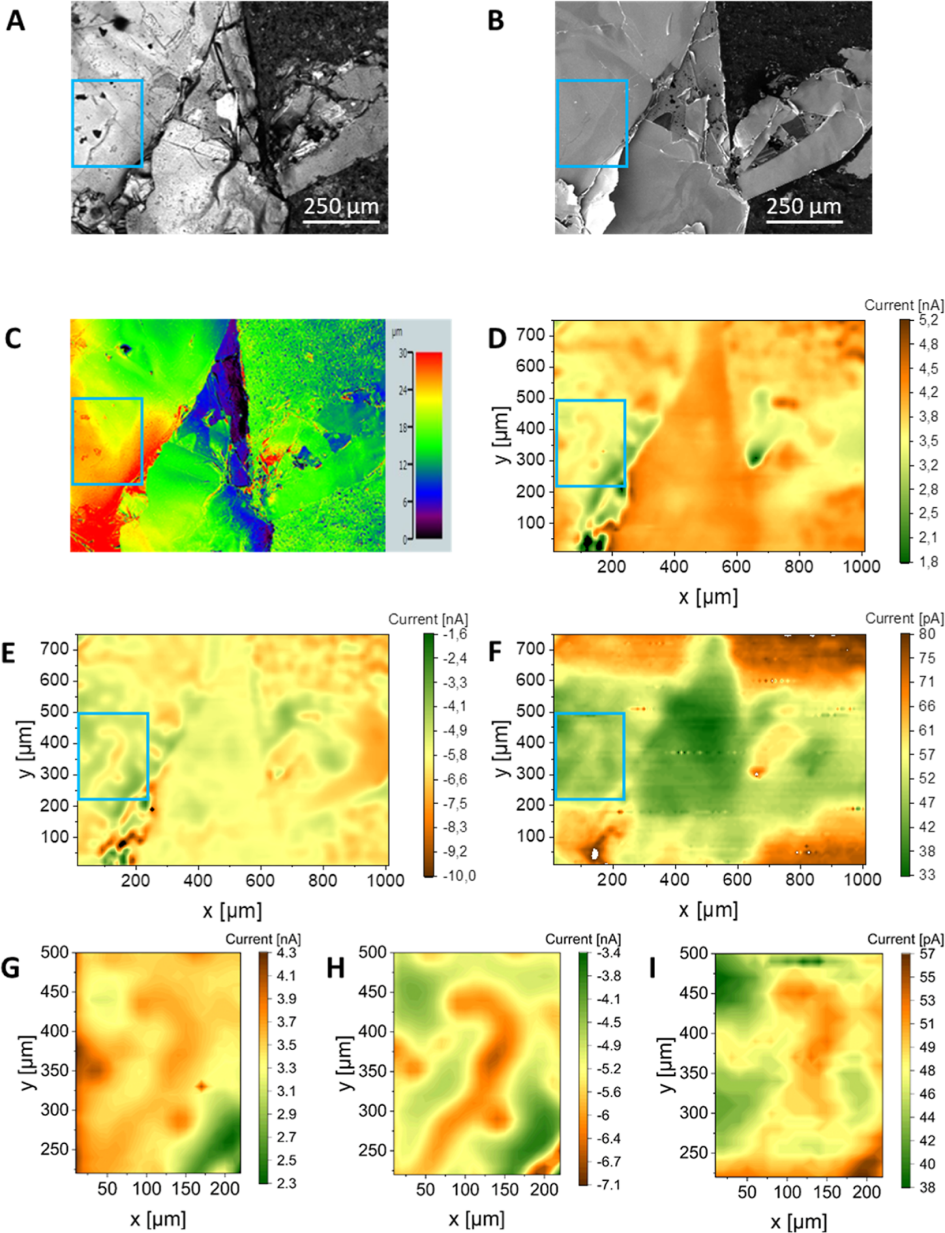
OM (A), SEM (B), CLSM (C), and SECM images of the FePSe_3_ sample (D–F). (D) Feedback SECM image recorded in 1.5 mM FcMeOH. *E*_probe_ = 0.5 V. (E) SG/TC SECM image recorded in 1.5 mM FcMeOH. *E*_probe_ = −0.2 V, *E*_substrate_ = 0.5 V. (F) SG/TC SECM image recorded in 0.5 M H_2_SO_4_. *E*_probe_ = 0.2 V, *E*_substrate_ = −0.5 V. Images (G–I) are extracted from the SECM images (D–F), highlighting the area included by the blue boxes. (G) Feedback SECM image. (H) SG/TC SECM image recorded in FcMeOH. (I) SG/TC SECM image recorded in H_2_SO_4_.

For correlating the local activity of the FePSe_3_ crystal, an OM image and an SEM image were recorded (Figure 5A+B). Because the topography of samples can impact the current measured in SECM, a CLSM image of the sample was recorded as well. The CLSM image in Figure 5C shows the height profile of the investigated area. The feedback mode image (Figure 5D), indicating the local conductivity of the sample, shows that the electron transport at the crystal surface is nonuniform. While the central piece of the crystal appears uniformly conductive, the left part shows local differences in conductivity (highlighted by a blue box, see Figure 5G for better visibility). In that region, the areas where higher currents were recorded are located where cracks (and thus crystal edges) are visible in the image in Figure 5A. Moreover, in the bottom left of the optical image, both low and high currents were measured by SECM. This high contrast in the SECM image indicates a low substrate-to-tip distance and that this piece of crystal protrudes from the sample surface. The CLSM image in Figure 5C proves that the crystal is protruding from the surface compared to the rest of the imaged area. The SG/TC SECM image in Figure 5E gives an insight into the local electrochemical activity of the FePSe_3_ crystal surface. The image shows well-pronounced local differences over the entirety of the investigated area. Especially in the left highlighted area (extracted in Figure 5H), the regions where a high current was recorded correlate to cracks visible in the optical image in Figure 5A. Because high currents in the SG/TC mode image indicate a high electrochemical activity, we can conclude that the edges of the crystal tend to be more electrochemically active than the basal planes. To investigate whether the electrocatalytic activity for the HER follows the same trend, another SG/TC mode image in 0.5 M H_2_SO_4_ was recorded. This resulted in an image showing local differences in the HER (Figure 5F). Relatively large current differences were located at the top and bottom borders of the image. Consequently, clearly distinguishing edge versus basal plane activity in these regions is not possible. In the left highlighted part (see Figure 5I for an extract of that area), however, more clear local differences in current were measured. Here, lines of high current, and thus high electrocatalytic activity, can be localized. They correspond to both electrochemically active regions (Figure 5D) and cracks within the sample (Figure 5A). Furthermore, the current pattern recorded in SECM images does not match the topography of the sample shown in Figure 5C, and consequently, these patterns of high currents are not caused by topographic effects. Thus, these results show that edges of FePSe_3_ are both more electrochemically and electrocatalytically active than the basal planes.

## Conclusions

In this work, we investigated whether the theoretical prediction that edges of MPX_3_ materials are more electroactive than their basal planes is valid. The local electrochemical investigation of an MPX_3_ representative, namely, FePSe_3_, via SECM has shown that edges exhibit both increased electrochemical and electrocatalytic activity toward the HER. Thus, our measurements indicate that the ideal MPX_3_-based electrocatalyst is rich in edge planes. This knowledge is very important for future catalyst design. It also adds MPX_3_ compounds to the family of materials where edges and defects are more active than the basal planes of 2D materials. Our work took a qualitative approach for basal plane and edge activity characterization. Thus, this study opens the door toward quantitative analysis of the activity of the basal planes and edges of MPX_3_ compounds. Another important question is whether the edges and basal planes of single layers of FePSe_3_ show similar characteristics as the bulk material analyzed in this study.

## Abbreviations

EDS: energy-dispersive X-ray spectroscopy
HER: hydrogen evolution reaction
LSV: linear scan voltammogram
OM: optical microscopy
PAC: probe approach curve
PDMS: polydimethylsiloxane
SECM: scanning electrochemical microscopy
SEM: scanning electron microscopy
SG/TC: substrate generation/tip collection

## References

[ref1] Wang M.; Zhang L.; He Y.; Zhu H. Recent advances in transition-metal-sulfide-based bifunctional electrocatalysts for overall water splitting. J. Mater. Chem. A 2021, 9, 5320–5363. 10.1039/D0TA12152E.

[ref2] Anasori B.; Lukatskaya M. R.; Gogotsi Y. 2D metal carbides and nitrides (MXenes) for energy storage. Nat. Rev. Mater. 2017, 2, 16098 10.1038/natrevmats.2016.98.

[ref3] Khan K.; Tareen A. K.; Aslam M.; Wang R.; Zhang Y.; Mahmood A.; Ouyang Z.; Zhang H.; Guo Z. Recent developments in emerging two-dimensional materials and their applications. J. Mater. Chem. C 2020, 8, 387–440. 10.1039/C9TC04187G.

[ref4] Novoselov K. S.; Geim A. K.; Morozov S. V.; Jiang D.; Zhang Y.; Dubonos S. V.; Grigorieva I. V.; Firsov A. A. Electric field effect in atomically thin carbon films. Science 2004, 306, 666–669. 10.1126/science.1102896. 15499015

[ref5] Gusmão R.; Sofer Z.; Bouša D.; Pumera M. Pnictogen (As, Sb, Bi) Nanosheets for Electrochemical Applications Are Produced by Shear Exfoliation Using Kitchen Blenders. Angew. Chem., Int. Ed. 2017, 56, 14417–14422. 10.1002/anie.201706389. 28755460

[ref6] Pumera M.; Sofer Z. 2D Monoelemental Arsenene, Antimonene, and Bismuthene: Beyond Black Phosphorus. Adv. Mater. 2017, 29, 1605299 10.1002/adma.201605299. 28185366

[ref7] Sturala J.; Sofer Z.; Pumera M. Chemistry of Layered Pnictogens: Phosphorus, Arsenic, Antimony, and Bismuth. Angew. Chem., Int. Ed. 2019, 58, 7551–7557. 10.1002/anie.201900811. 30994978

[ref8] Beladi-Mousavi S. M.; Pumera M. 2D-Pnictogens: alloy-based anode battery materials with ultrahigh cycling stability. Chem. Soc. Rev. 2018, 47, 6964–6989. 10.1039/C8CS00425K. 30177984

[ref9] Chia X.; Eng A. Y. S.; Ambrosi A.; Tan S. M.; Pumera M. Electrochemistry of Nanostructured Layered Transition-Metal Dichalcogenides. Chem. Rev. 2015, 115, 11941–11966. 10.1021/acs.chemrev.5b00287. 26426313

[ref10] Chia X.; Pumera M. Characteristics and performance of two-dimensional materials for electrocatalysis. Nat. Catal. 2018, 1, 909–921. 10.1038/s41929-018-0181-7.

[ref11] Eng A. Y. S.; Ambrosi A.; Sofer Z.; Šimek P.; Pumera M. Electrochemistry of transition metal dichalcogenides: strong dependence on the metal-to-chalcogen composition and exfoliation method. ACS Nano 2014, 8, 12185–12198. 10.1021/nn503832j. 25453501

[ref12] Toh R. J.; Sofer Z.; Pumera M. Catalytic properties of group 4 transition metal dichalcogenides (MX2; M = Ti, Zr, Hf; X = S, Se, Te). J. Mater. Chem. A 2016, 4, 18322–18334. 10.1039/C6TA08089H.

[ref13] Wang Z.; Wu H.-H.; Li Q.; Besenbacher F.; Li Y.; Zeng X. C.; Dong M. Reversing Interfacial Catalysis of Ambipolar WSe 2 Single Crystal. Adv. Sci. 2020, 7, 1901382 10.1002/advs.201901382. PMC7001631 32042552

[ref14] Hu F.; Yu D.; Ye M.; Wang H.; Hao Y.; Wang L.; Li L.; Han X.; Peng S. Lattice-Matching Formed Mesoporous Transition Metal Oxide Heterostructures Advance Water Splitting by Active Fe-O-Cu Bridges. Adv. Energy Mater. 2022, 12, 2200067 10.1002/aenm.202200067.

[ref15] Huang H.; Yu D.; Hu F.; Huang S.-C.; Song J.; Chen H.-Y.; Li L. L.; Peng S. Clusters Induced Electron Redistribution to Tune Oxygen Reduction Activity of Transition Metal Single-Atom for Metal-Air Batteries. Angew. Chem., Int. Ed. 2022, 61, e202116068 10.1002/anie.202116068. 34957659

[ref16] Deng L.; Hu F.; Ma M.; Huang S.-C.; Xiong Y.; Chen H.-Y.; Li L.; Peng S. Electronic Modulation Caused by Interfacial Ni-O-M (M=Ru, Ir, Pd) Bonding for Accelerating Hydrogen Evolution Kinetics. Angew. Chem., Int. Ed. 2021, 60, 22276–22282. 10.1002/anie.202110374. 34427019

[ref17] Kang Z.; Khan M. A.; Gong Y.; Javed R.; Xu Y.; Ye D.; Zhao H.; Zhang J. Recent progress of MXenes and MXene-based nanomaterials for the electrocatalytic hydrogen evolution reaction. J. Mater. Chem. A 2021, 9, 6089–6108. 10.1039/D0TA11735H.

[ref18] Seh Z. W.; Fredrickson K. D.; Anasori B.; Kibsgaard J.; Strickler A. L.; Lukatskaya M. R.; Gogotsi Y.; Jaramillo T. F.; Vojvodic A. Two-Dimensional Molybdenum Carbide (MXene) as an Efficient Electrocatalyst for Hydrogen Evolution. ACS Energy Lett. 2016, 1, 589–594. 10.1021/acsenergylett.6b00247.

[ref19] Qin T.; Wang Z.; Wang Y.; Besenbacher F.; Otyepka M.; Dong M. Recent Progress in Emerging Two-Dimensional Transition Metal Carbides. Nano-Micro Lett. 2021, 13, 183 10.1007/s40820-021-00710-7. PMC8379312 34417663

[ref20] Muller G. A.; Cook J. B.; Kim H.-S.; Tolbert S. H.; Dunn B. High performance pseudocapacitor based on 2D layered metal chalcogenide nanocrystals. Nano Lett. 2015, 15, 1911–1917. 10.1021/nl504764m. 25654445

[ref21] Ghosh K.; Pumera M. Free-standing electrochemically coated MoSx based 3D-printed nanocarbon electrode for solid-state supercapacitor application. Nanoscale 2021, 13, 5744–5756. 10.1039/D0NR06479C. 33724279

[ref22] Liu Y.; Wu J.; Hackenberg K. P.; Zhang J.; Wang Y. M.; Yang Y.; Keyshar K.; Gu J.; Ogitsu T.; Vajtai R.; Lou J.; Ajayan P. M.; Wood B. C.; Yakobson B. I. Self-optimizing, highly surface-active layered metal dichalcogenide catalysts for hydrogen evolution. Nat. Energy 2017, 2, 17127 10.1038/nenergy.2017.127.

[ref23] Li L.; Yu D.; Li P.; Huang H.; Xie D.; Lin C.-C.; Hu F.; Chen H.-Y.; Peng S. Interfacial electronic coupling of ultrathin transition-metal hydroxide nanosheets with layered MXenes as a new prototype for platinum-like hydrogen evolution. Energy Environ. Sci. 2021, 14, 6419–6427. 10.1039/D1EE02538D.

[ref24] Samal R.; Sanyal G.; Chakraborty B.; Rout C. S. Two-dimensional transition metal phosphorous trichalcogenides (MPX3): a review on emerging trends, current state and future perspectives. J. Mater. Chem. A 2021, 9, 2560–2591. 10.1039/D0TA09752G.

[ref25] Mayorga-Martinez C. C.; Sofer Z.; Sedmidubský D.; Huber Š.; Eng A. Y. S.; Pumera M. Layered Metal Thiophosphite Materials: Magnetic, Electrochemical, and Electronic Properties. ACS Appl. Mater. Interfaces 2017, 9, 12563–12573. 10.1021/acsami.6b16553. 28355055

[ref26] Gusmão R.; Sofer Z.; Pumera M. Exfoliated Layered Manganese Trichalcogenide Phosphite (MnPX 3 , X = S, Se) as Electrocatalytic van der Waals Materials for Hydrogen Evolution. Adv. Funct. Mater. 2019, 29, 1805975 10.1002/adfm.201805975.

[ref27] Gusmão R.; Sofer Z.; Sedmidubský D.; Huber Š.; Pumera M. The Role of the Metal Element in Layered Metal Phosphorus Triselenides upon Their Electrochemical Sensing and Energy Applications. ACS Catal. 2017, 7, 8159–8170. 10.1021/acscatal.7b02134.

[ref28] Sanna M.; Ng S.; Pumera M. Layered transition metal selenophosphites for visible light photoelectrochemical production of hydrogen. Electrochem. Commun. 2021, 129, 107077 10.1016/j.elecom.2021.107077.

[ref29] Barua M.; Ayyub M. M.; Vishnoi P.; Pramoda K.; Rao C. N. R. Photochemical HER activity of layered metal phospho-sulfides and -selenides. J. Mater. Chem. A 2019, 7, 22500–22506. 10.1039/C9TA06044H.

[ref30] Song J. Sub-2 nm Thiophosphate Nanosheets with Heteroatom Doping for Enhanced Oxygen Electrocatalysis. Adv. Funct. Mater. 2021, 31, 2100618 10.1002/adfm.202100618.

[ref31] Banks C. E.; Davies T. J.; Wildgoose G. G.; Compton R. G. Electrocatalysis at graphite and carbon nanotube modified electrodes: edge-plane sites and tube ends are the reactive sites. Chem. Commun. 2005, 829–841. 10.1039/b413177k. 15700054

[ref32] Wang L.; Sofer Z.; Pumera M. Will Any Crap We Put into Graphene Increase Its Electrocatalytic Effect?. ACS Nano 2020, 14, 21–25. 10.1021/acsnano.9b00184. 31934742

[ref33] Güell A. G.; Cuharuc A. S.; Kim Y.-R.; Zhang G.; Tan S.; Ebejer N.; Unwin P. R. Redox-dependent spatially resolved electrochemistry at graphene and graphite step edges. ACS Nano 2015, 9, 3558–3571. 10.1021/acsnano.5b00550. 25758160

[ref34] Yuan W.; Zhou Y.; Li Y.; Li C.; Peng H.; Zhang J.; Liu Z.; Dai L.; Shi G. The edge- and basal-plane-specific electrochemistry of a single-layer graphene sheet. Sci. Rep. 2013, 3, 2248 10.1038/srep02248. 23896697 PMC3727060

[ref35] Bentley C. L.; Kang M.; Maddar F. M.; Li F.; Walker M.; Zhang J.; Unwin P. R. Electrochemical maps and movies of the hydrogen evolution reaction on natural crystals of molybdenite (MoS2): basal vs. edge plane activity. Chem. Sci. 2017, 8, 6583–6593. 10.1039/c7sc02545a. 28989686 PMC5627349

[ref36] Jaramillo T. F.; Jørgensen K. P.; Bonde J.; Nielsen J. H.; Horch S.; Chorkendorff I. Identification of Active Edge Sites for Electrochemical H 2 Evolution from MoS 2 Nanocatalysts. Science 2007, 317, 100–102. 10.1126/science.1141483. 17615351

[ref37] Takahashi Y.; Kobayashi Y.; Wang Z.; Ito Y.; Ota M.; Ida H.; Kumatani A.; Miyazawa K.; Fujita T.; Shiku H.; Korchev Y. E.; Miyata Y.; Fukuma T.; Chen M.; Matsue T. High-Resolution Electrochemical Mapping of the Hydrogen Evolution Reaction on Transition-Metal Dichalcogenide Nanosheets. Angew. Chem., Int. Ed. 2020, 59, 3601–3608. 10.1002/anie.201912863. 31777142

[ref38] Tan S. M.; Ambrosi A.; Sofer Z.; Huber Š.; Sedmidubský D.; Pumera M. Pristine Basal- and Edge-Plane-Oriented Molybdenite MoS2Exhibiting Highly Anisotropic Properties. Chemistry 2015, 21, 7170–7178. 10.1002/chem.201500435. 25821017

[ref39] Tao B.; Unwin P. R.; Bentley C. L. Nanoscale Variations in the Electrocatalytic Activity of Layered Transition-Metal Dichalcogenides. J. Phys. Chem. C 2020, 124, 789–798. 10.1021/acs.jpcc.9b10279.

[ref40] Wert S.; Iffelsberger C.; Novčić K. A.; Matysik F.-M.; Pumera M. Edges are more electroactive than basal planes in synthetic bulk crystals of TiS2 and TiSe2. Appl. Mater. Today 2022, 26, 101309 10.1016/j.apmt.2021.101309.

[ref41] Sofer Z.; Sedmidubský D.; Huber Š.; Luxa J.; Bouša D.; Boothroyd C.; Pumera M. Layered Black Phosphorus: Strongly Anisotropic Magnetic, Electronic, and Electron-Transfer Properties. Angew. Chem., Int. Ed. 2016, 55, 3382–3386. 10.1002/anie.201511309. 26822395

[ref42] Marvan P.; Huber Š.; Luxa J.; Mazánek V.; Sedmidubský D.; Sofer Z.; Pumera M. Edge vs. basal plane electrochemistry of layered pnictogens (As, Sb, Bi): Does edge always offer faster electron transfer?. Appl. Mater. Today 2019, 16, 179–184. 10.1016/j.apmt.2019.05.009.

[ref43] Djire A.; Wang X.; Xiao C.; Nwamba O. C.; Mirkin M. V.; Neale N. R. Basal Plane Hydrogen Evolution Activity from Mixed Metal Nitride MXenes Measured by Scanning Electrochemical Microscopy. Adv. Funct. Mater. 2020, 30, 2001136 10.1002/adfm.202001136.

[ref44] Das T.; Alam K.; Chakraborty S.; Sen P. Probing active sites on MnPSe3 and FePSe3 tri-chalcogenides as a design strategy for better hydrogen evolution reaction catalysts. Int. J. Hydrogen Energy 2021, 46, 37928–37938. 10.1016/j.ijhydene.2021.09.074.

[ref45] Bentley C. L.; Edmondson J.; Meloni G. N.; Perry D.; Shkirskiy V.; Unwin P. R. Nanoscale Electrochemical Mapping. Anal. Chem. 2019, 91, 84–108. 10.1021/acs.analchem.8b05235. 30500157

[ref46] https://www.2dsemiconductors.com/FePSe3/#description, accessed Aug 30, 2022.

